# Small Interfering RNA Targeted to IGF-IR Delays Tumor Growth and Induces Proinflammatory Cytokines in a Mouse Breast Cancer Model

**DOI:** 10.1371/journal.pone.0029213

**Published:** 2012-01-03

**Authors:** Tiphanie Durfort, Mercedes Tkach, Mariya I. Meschaninova, Martín A. Rivas, Patricia V. Elizalde, Alya G. Venyaminova, Roxana Schillaci, Jean-Christophe François

**Affiliations:** 1 Institut National de la Santé et de la Recherche Médicale (INSERM) U565, Paris, France; 2 Centre National de la Recherche, Scientifique, UMR 7196; Muséum National d'Histoire Naturelle, Paris, France; 3 Instituto de Biología y Medicina Experimental (IBYME), Consejo de Investigaciones Científicas y Técnicas (CONICET), Buenos Aires, Argentina; 4 Institute of Chemical Biology and Fundamental Medicine - Siberian Division of Russian Academy of Sciences (SB-RAS), Novosibirsk, Russia; University of Florida, United States of America

## Abstract

Insulin-like growth factor I (IGF-I) and its type I receptor (IGF-IR) play significant roles in tumorigenesis and in immune response. Here, we wanted to know whether an RNA interference approach targeted to IGF-IR could be used for specific antitumor immunostimulation in a breast cancer model. For that, we evaluated short interfering RNA (siRNAs) for inhibition of *in vivo* tumor growth and immunological stimulation in immunocompetent mice. We designed 2′-O-methyl-modified siRNAs to inhibit expression of IGF-IR in two murine breast cancer cell lines (EMT6, C4HD). Cell transfection of IGF-IR siRNAs decreased proliferation, diminished phosphorylation of downstream signaling pathway proteins, AKT and ERK, and caused a G0/G1 cell cycle block. The IGF-IR silencing also induced secretion of two proinflammatory cytokines, TNF- α and IFN-γ. When we transfected C4HD cells with siRNAs targeting IGF-IR, mammary tumor growth was strongly delayed in syngenic mice. Histology of developing tumors in mice grafted with IGF-IR siRNA treated C4HD cells revealed a low mitotic index, and infiltration of lymphocytes and polymorphonuclear neutrophils, suggesting activation of an antitumor immune response. When we used C4HD cells treated with siRNA as an immunogen, we observed an increase in delayed-type hypersensitivity and the presence of cytotoxic splenocytes against wild-type C4HD cells, indicative of evolving immune response. Our findings show that silencing IGF-IR using synthetic siRNA bearing 2′-O-methyl nucleotides may offer a new clinical approach for treatment of mammary tumors expressing IGF-IR. Interestingly, our work also suggests that crosstalk between IGF-I axis and antitumor immune response can mobilize proinflammatory cytokines.

## Introduction

Insulin-like growth factor type I receptor (IGF-IR) signaling has a significant impact on development of many normal tissues, and also on growth of malignant tumors [1]. Epidemiological studies showed that increased serum concentration of insulin-like growth factor I (IGF-I) is associated with increased risk of developing tumors including those of the breast [2]. Moreover, IGF-IR is a potent control point for transformation and is therefore considered as a relevant therapeutic target [3]. Indeed, drugs targeting the IGF axis are under development by major companies and include receptor-specific blocking antibodies and tyrosine kinase inhibitors (TKIs) [4]. Other approaches using nucleic-acid based strategies have been used to investigate the IGF-IR/IGF-I pathway, including antisense oligonucleotides, antisense RNA expression plasmids, ribozymes, triplex-forming oligonucleotides and short interfering RNAs (siRNAs) [5], [6], [7], [8], [9], [10]. Although nucleic-acid based approaches are theoretically specific and selective, they may have the undesirable effect of silencing non-targeted mRNAs, more particularly in the case of siRNAs and phosphorothioate antisense oligonucleotides [11].

It was previously found that down-regulation of IGF-IR using antisense expression vectors may block tumor growth *in vivo* [12], [13], [14], [15] For example, murine EMT6 breast cancer cells carrying an antisense IGF-IR vector exhibited a significant decrease in cell proliferation *in vitro*, lost their ability to form colonies in soft agar, and also lost their tumorigenic property when grafted to syngenic mice [16]. Interestingly, antisense down-regulation of IGF-IR can unexpectedly induce an antitumor host response with several of the characteristics of an immune response. Injection of glioblastoma cells stably expressing antisense IGF-IR transcripts in syngenic rats elicited a protective host response that inhibited tumor formation by subsequent injection of wild-type cells associated to the proliferation of cytotoxic CD8+ lymphocytes [17]. This host immune response was also observed in experiments with other syngenic models such as mouse neuroblastoma or melanoma [13], [18]. Although few attempts were done to unravel the mechanisms leading to this host response after IGF-IR down-regulation, it has been hypothesized that this could be due to induction of *in vivo* apoptosis or to secretion of immuno-peptides that interact with Major Histocompatibility Complex (MHC) class I antigen, further recognized by CD8+ cells [18], [19], [20].

We have shown that *in vivo* administration of phosphorothioate antisense oligonucleotides targeting IGF-IR decreased receptor protein levels and concomitantly inactivated AKT and MAPK signaling pathways leading to C4HD breast tumor growth inhibition [21]. We successfully protected syngenic mice from tumor development induced by wild-type C4HD by inoculating mice with C4HD cells treated with antisense oligonucleotides targeting IGF-IR [22]. Similarly tumor-specific immunity led to inhibition of tumor growth through the generation of a cellular response and of tumor-specific cytotoxic cells. Down-regulation of IGF-IR up-regulated the co-stimulatory molecule CD86 as well as the peptide-chaperone Hsp70 [22]. A significant body of evidence indicates that the IGF-I/IGF-IR axis interferes with immune recognition of tumor cells [13], [17], [23]. Indeed, triple helix-forming or antisense expression vectors targeting IGF-I induced a host immune response with up-regulation of immunogenic molecules and increased production of apoptotic cells [23], [24], [25]

Here, we analyzed the effect of transiently silencing of IGF-IR into mouse breast cancer cells through transfection of well-defined small molecules, such as siRNAs modified with 2′-O-methyl nucleotides for *in vivo* use. These short molecules are supposed to be more specific than antisense RNA and devoid of undesired effects [11]. Using the most efficient siRNAs, we inhibited IGF-IR downstream signaling proteins, and confirmed its essential role for *in vitro* cell growth and cell cycle regulation. Remarkably, blocking IGF-IR signaling in breast cancer cells not only decreased tumor growth in syngenic mice and triggered features of an immune response, but also induced secretion of pro-inflammatory cytokines. These results are strong evidence for significant links between IGF-IR and immune response pathways.

## Materials and Methods

### Ethics Statements

Animal studies were conducted as in accordance with the highest standards of animal care as outlined by the NIH Guide for the Care and Use of Laboratory Animals [26], and were approved by the IBYME Animal Research Committee. The IBYME is approved by OLAW, NIH (assurance #A5072-01).

### Reagents

All chemical reagents were from Sigma-Aldrich, (St. Louis, MO), unless otherwise indicated. Rabbit polyclonal anti human IGF-IR ß (C-20) antibody, mouse monoclonal anti human INS-R ß (9H4) antibody and mouse monoclonal anti rabbit GAPDH (6C5) antibody were from Santa Cruz Biotechnology, Santa Cruz, CA. Rabbit monoclonal anti human phospho-p44/42 MAPK (Thr202/Tyr204) antibody, rabbit polyclonal anti rat p44/42 MAPK (Erk1/2) antibody, rabbit polyclonal anti mouse phospho AKT (Ser473) antibody, rabbit polyclonal anti mouse AKT antibody were from Cell Signaling Technology, Beverly, MA. Mouse monoclonal anti ß-actin (A5316) was from Sigma-Aldrich. Sheep anti-mouse IgG peroxidase-linked whole antibody (NXA931) and donkey anti-rabbit IgG peroxidase-linked whole antibody (NA934) were from GE Healthcare, Little Chalfont, UK.

### Animals and tumors

Experiments were carried out in virgin female BALB/c mice raised at the IBYME. The hormone-dependent ductal tumor line C4HD was originated in mice treated with 40 mg medroxyprogesterone acetate (MPA, medrosterona, Laboratorios Gador, Buenos Aires, Argentina) every 3 months for 1 year, and has been maintained by serial transplantation in animals treated with 40 mg subcutaneously (s.c.) MPA depot in the opposite flank to tumor inoculum [21], [27]. The C4HD tumor line is of ductal origin, expresses progesterone and estrogen receptors, IGF-I/IGF-IR, lacks glucocorticoid receptor expression and requires MPA administration to proliferate both *in vivo* and *in vitro* [21].

### Cell lines and culture

The EMT6 murine mammary carcinoma cells were purchased from American Type Culture Collection (CRL-2755) and were maintained in Waymouth's MB 752/1 medium (Invitrogen, Cergy-Pontoise, France) supplemented with 10% heat-inactivated fetal calf serum (FBS; Hyclone, Thermofisher, Strasbourg, France) and 2 mM L-glutamine (Invitrogen). EMT6 cells express large amount of IGF-IR [16]. All cell lines were regularly checked for mycoplasma infection using PCR (Venor®GeM, Biovalley, Marne-la-Vallée, France). Primary cultures of epithelial cells from C4HD tumors, growing in MPA-treated mice, were performed as described [28], [29]. Epithelial cells were plated in flasks with DMEM/F12+5% steroid-stripped FCS (ChFCS, Gen S.A., Buenos Aires), and allowed to attach for 24 h. Purity of epithelial cultures was evaluated by cytokeratin staining. Cells were incubated in DMEM/F12, (100 U/ml penicillin, 100 µg/ml streptomycin, without phenol red), with 2.5% ChFCS and 10 nM MPA.

### SiRNA and cell transfection

The synthesized siRNAs (nomenclature and design in Methods S1) were transfected into EMT6 cells using a reverse transfection procedure with Lipofectamine™ RNAiMAX (Invitrogen, Cergy-Pontoise, France). DharmaFECT-I cationic lipid (Thermo Fisher Scientific, USA) was used for siRNA transfection into C4HD cells using a direct transfection protocol. After transfection, cells were incubated at 37°C for 24 h, to 72 h before further analysis. RNA preparation, quantitative RT-PCR, gel electrophoresis and immunobloting procedures are described in Methods S1.

### Proliferation and cell cycle assays

Cell proliferation was evaluated 48 h post-transfection with siRNAs targeting IGF-IR using CellTiter 96 AQueous Non-Radioactive Cell Proliferation Assay (Promega, Charbonnières, France). Cell proliferation assays performed in triplicate using three independent transfections were reproduced in two independent experiments. Cell-cycle analysis was done using propidium iodide (2.5 mg/mL) staining of methanol-fixed samples treated with RNase (100 µg/mL). All data were analyzed with Dean-Jett-Fox model cell cycle analysis using FlowJo 8.8.6 software (Tree Star, Inc., Olten, Switzerland) including doublet discrimination.

### *In vivo* tumor growth experiments

C4HD cells growing in MPA (10 nM) were transiently transfected with 2′-O-methyl- modified siRNA (100 nM) using Dharmafect-I (Thermo Fisher Scientific, USA). After 48 h, 2×10^6^ cells were inoculated s.c. in BALB/c females treated with 40 mg MPA depot in the flank opposite to the cell inoculums (n = 5 per group). Tumor volume and growth rate were determined as described [22]. At day 26, animals were euthanized and tumors removed. Tissues were fixed in 10% buffered formalin and embedded in paraffin; 5 µm sections were stained with hematoxylin and eosin (H&E) for microscopy. Immunization of mice with transfected cells was described in Methods S1.

### Mouse cytokine antibody array

Conditioned media were harvested from untreated C4HD cells or siRNA-transfected C4HD cells grown in DMEM/F12 with 2.5% ChFCS and 10 nM MPA for 48 h. Mouse cytokine antibody arrays (Panomics, Redwood City, CA) were used to profile cytokines produced by 2 ml of conditioned media. This experiment was performed twice. Graphs correspond to densitometric analysis of the chemiluminescent signal as described in Methods S1.

### Statistical analysis

Cell data were derived from at least two independent experiments, each with three independent transfection assays. Statistical analyses were conducted using Prism 5.0a GraphPad software. Comparisons among groups were performed with one-way analysis of variance test. If statistically significant, the Dunnett's multiple comparison *post hoc* test was used. Values of *P*<0.05 were considered significant and indicated asterisks refer to comparison to samples transfected with control siRNA. Data are presented as the mean ± SEM [30]. Where indicated, Student's *t*-test was also used for comparison. For *in vivo* studies, comparison of tumor volumes between the different groups was done by ANOVA followed by Tukey *post hoc* test. Linear regression analysis was performed on tumor growth curves, and the slopes were compared using ANOVA followed by parallelism test to evaluate the statistical significance of the differences. Values of *P*<0.05 were considered significant.

## Results

### Identification of mouse IGF-IR siRNAs

**Figure 1:**
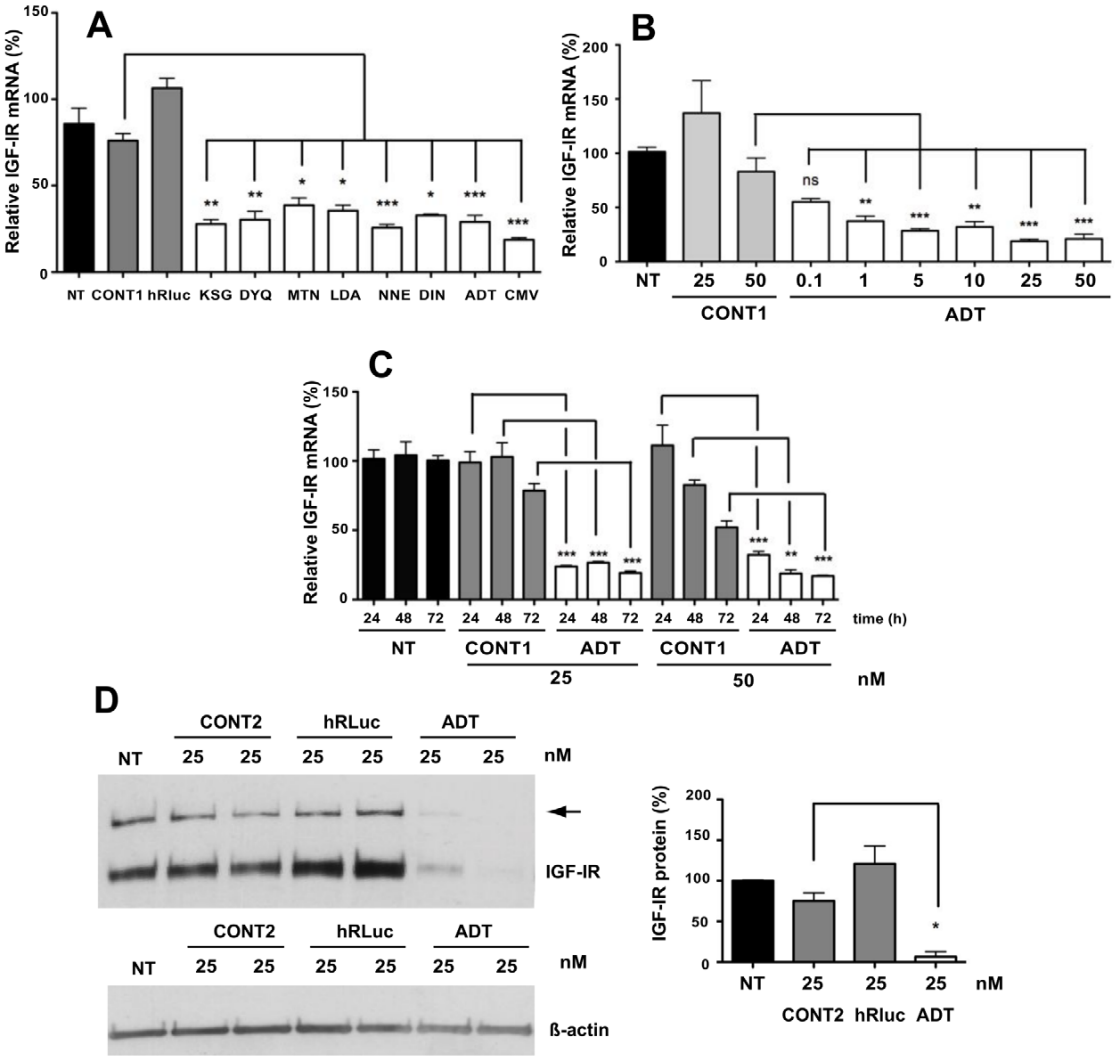
siRNAs targeted to mouse IGF-IR in breast cancer cells. (**A**) The effect of siRNAs (10 nM) designed against IGF-IR coding regions was measured by qRT-PCR 24 h post-transfection in EMT6 cells. (**B**) Dose-dependent inhibition of IGF-IR mRNA levels by ADT siRNA into EMT6. (**C**) Kinetics of silencing by ADT siRNA in EMT6 cells. Black bars represent mock transfected cells (NT). Means ± SEM of two independent experiments with independent transfected quadruplicates were shown; * *P*<0.05; ** *P*<0.01; *** *P*<0.001. Significant inhibition of IGF-IR mRNA by ADT persisted for 72 h at both concentrations, whereas 50 nM of CONT1 started to decrease significantly IGF-IR mRNA at 72 h when compared to untreated cells (*P*<0.001). (**D**) Western blot analysis after 48 h transfection of EMT6 cells with unmodified siRNAs. Arrows indicate the 200 kDa IGF-IR proreceptor. NT and black bars are non-transfected cells. Representative Western blot is presented and quantitative analysis of three independent experiments is shown next to the gel with means ± SEM. * *P*<0.05.

As shown by qRT-PCR, all eight siRNA targeted to mouse IGF-IR (mIGF-IR) (siRNA sequences in Table S1) efficiently inhibited production of IGF-IR mRNA in mouse breast cancer EMT6 cells [16]. KSG, DYQ, NNE, ADT and CMV siRNAs inhibited IGF-IR mRNA expression by approximately 70% compared to untreated cells and to cells transfected with control siRNAs (Figure 1A). After careful examination of all siRNAs for their GC content, their potential secondary structures and the presence of potential immunostimulatory motifs, ADT siRNA was selected for further evaluation [31]. Dose-response experiments performed with ADT siRNA in EMT6 cells showed that 50 nM of ADT decreased IGF-IR mRNA by 83% compared to levels in untreated cells (Figure 1B). Furthermore, kinetic experiments demonstrated that increasing incubation time after transfection improved silencing efficiency of anti IGF-IR siRNA (Figure 1C). The observed IGF-IR silencing remains specific even though prolonged exposure of cells with high concentration of siRNAs and cationic lipids led to a decrease in IGF-IR mRNA levels, as shown in Figure 1C with 50 nM CONT1 72 h post-transfection. Western blot analysis revealed an efficient down-regulation of endogenous IGF-IR expression in ADT-transfected EMT6 cells (94% at 25 nM; Figure 1D).

**Figure 2:**
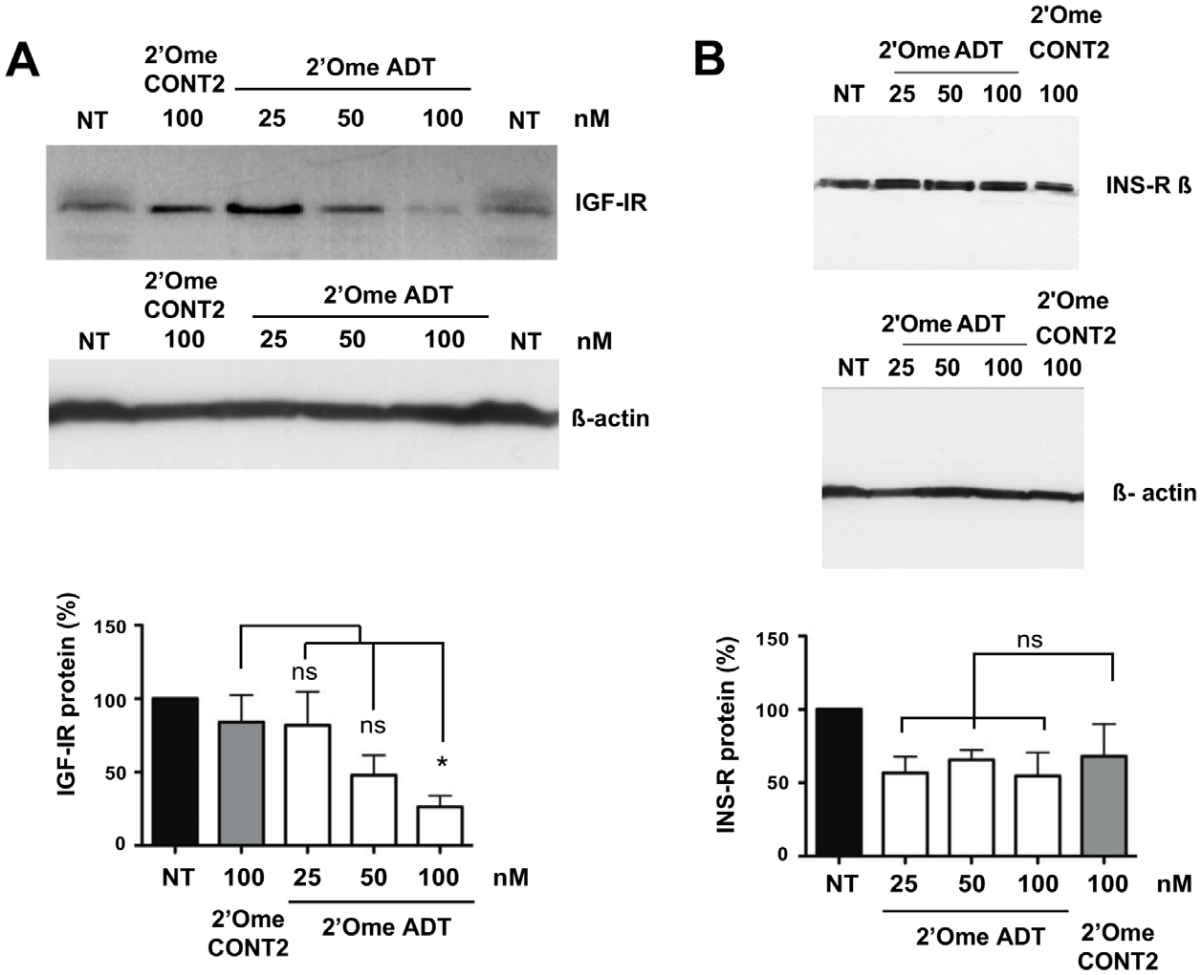
2′-O-methyl siRNAs targeted to IGF-IR. (A), Western blot analysis of IGF-IR after 48 h transfection of EMT6 cells with 2′-O-methyl siRNAs. NT and black bars are non-transfected cells. Western blot example and quantitative analysis of five independent experiments are shown with means ± SEM. * P<0.05; ns, not significant. (B), Western blot analysis of INS-R proteins isolated from EMT6 cells 48 h after transfection with 2′-O-methyl uridine containing anti IGF-IR siRNAs at 25, 50 or 100 nM. Black bars represent mock transfected cells (NT). A representative experiment is presented with quantitative analysis of four independent experiments (means ± SEM; ns, not significant). Differences between NT and siRNA treated samples were not statistically significant.

Although unmodified siRNAs worked well in mouse breast cancer cells, a higher stability is required for *in vivo* applications. A range of oligonucleotide modifications confers nuclease resistance to siRNAs such as 2′-O-methyl modification [31], [32]. Moreover, while unmodified siRNAs were known to produce non-specific immunostimulatory effects due to sequence motifs [31], we decided to substitute few uridine residues in siRNA sense strand by 2′-O-methyl uridines to get efficient silencing without immunostimulation non-related to IGF-IR silencing (Table S1). The introduction of these two modifications partially attenuated silencing of IGF-IR in EMT6 cells (Figure 2A). A detectable reduction in efficacy of the modified siRNA was observed when 25 nM of ADT and 25 nM of 2′-O-methyl ADT siRNAs were compared (Figure 1D and 2A). To characterize the specificity of the 2′-O-methyl ADT siRNA at high concentration (100 nM), we measured the levels of insulin receptor (INS-R) that is highly homologous to IGF-IR (70% amino acid identity), especially in the tyrosine kinase domain, in which they share 84% amino acid identity (Figure S1). Western blot analysis revealed that transfection of 2′-O-methyl ADT siRNA did not induce significant down-regulation of INS-R when compared to 2′-O-methyl CONT2 treatment (Figure 2B).

### IGF-IR inhibition blocks downstream signaling, induces cell-cycle arrest and decreases cell proliferation

**Figure 3:**
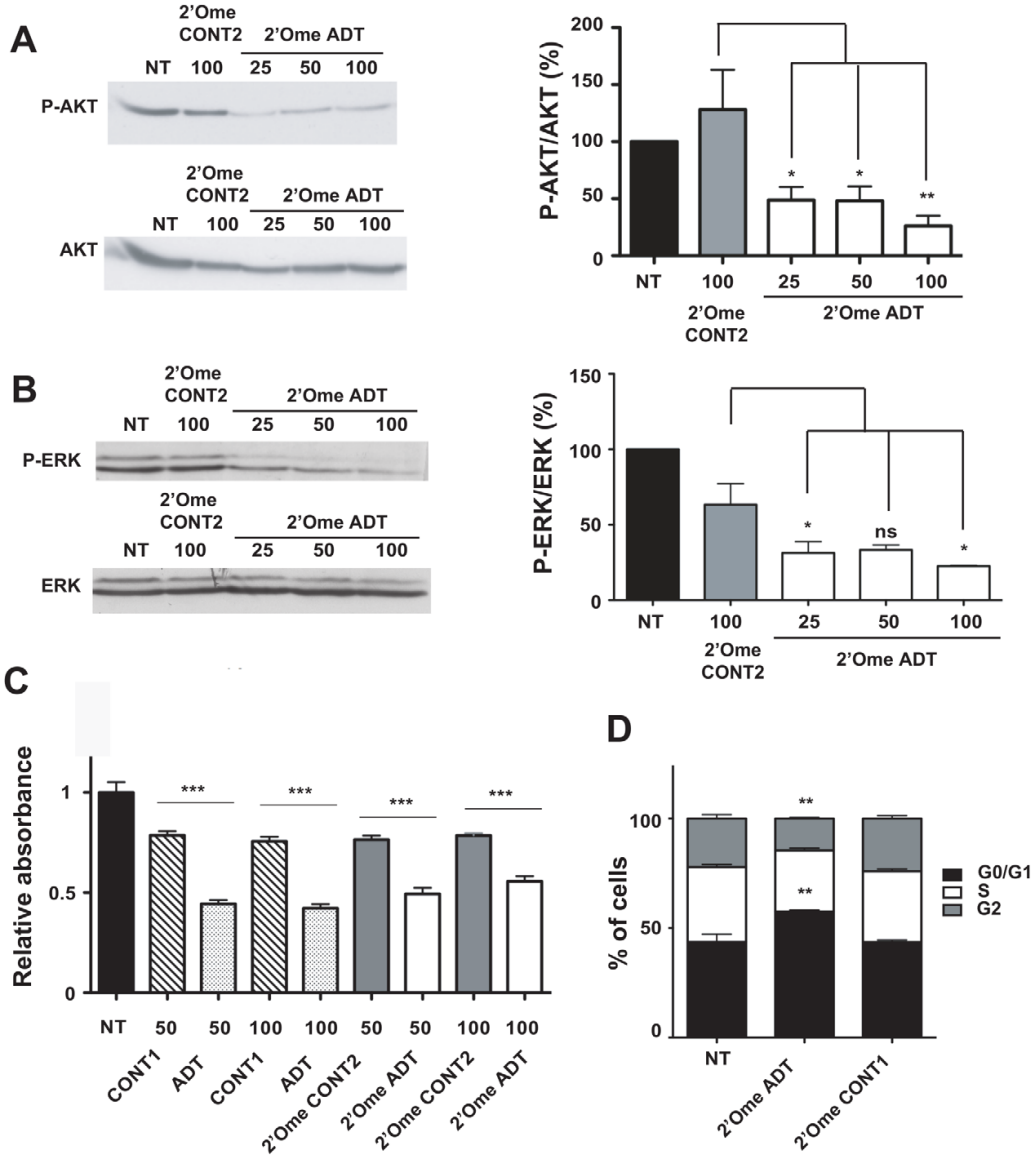
IGF-IR gene silencing inhibits signaling, cell cycle progression and proliferation. EMT6 cells were transfected with 25 to 100 nM 2′-O-methyl ADT siRNA and harvested 48 h later after standard growth conditions (10% FCS) for analysis by immunoblotting with: (**A**) antibodies against phospho-S473 AKT and total AKT, and (**B**) antibodies against phospho-T202/T204 ERKs and total ERK. Black bars represent mock transfected cells (NT). Representative experiments are presented and quantitative analysis of three independent experiments are shown on the right of each gel with means ± SEM. * *P*<0.05; ns, not significant. (**C**) Proliferation rates of siRNA-transfected EMT6 cells 48 h post-transfection; *** *P*<0.001, Student's t-test. (**D**) For cell cycle analysis, EMT6 cells were transfected with 50 nM siRNA and analyzed 48 h later by cytometry and propidium iodide staining. Experiments were performed in triplicate. Statistical analysis was referred to control siRNA treated cells. ** *P*<0.01.

In EMT6 cells, there was a clear inhibition of IGF-IR protein levels 48 h after transfection with 2′-O-methyl ADT siRNA at concentrations of 50 nM and above. Transfected cells were grown under standard growth conditions (10% FCS) before AKT/PKB and ERK1/2 activities were assessed by immunoblotting with phospho-specific antibodies. IGF-IR knockdown induced inhibition of AKT phosphorylation, as previously described using either unmodified siRNA or antisense phosphorothioate oligonucleotides in other cell lines (Figure 3A) [21], [33]. ERK phosphorylation was also inhibited in breast cancer cells when they were treated with 2′-O-methyl ADT siRNA (Figure 3B). Total AKT/PKB and ERK1/2 protein levels remain constant whatever siRNA was transfected. In our assay conditions, downregulation of p-ERK1/2 and p-AKT/PKB started to reach maximal efficiency at low concentration of siRNA. For *in vivo* analysis, the highest concentration of siRNA was chosen to maximize effect of IGF-IR silencing on signaling. To assess the effect of IGF-IR down-regulation on their growth, EMT6 were transfected with siRNAs and growth rate was analyzed using a colorimetric MTS-based assay (Figure 3C). Unmodified and 2′-O-methyl ADT transfection resulted in a 40–50% decreased cell growth rate at 50–100 nM as compared to cells transfected with control siRNA. To further elucidate how IGF-IR suppression affects cell proliferation, the cell-cycle status of siRNA-transfected cells was determined. Flow cytometric cell cycle analysis indicated that treatment with 100 nM 2′-O-methyl ADT arrested EMT6 in the G0/G1 phase of the cell cycle. The proportion of cells in the S- and G2-phases were decreased relative to those in cells treated with control siRNA (Figure 3D). Moreover, no sub G1 peak was detected with 100 nM 2′-O-methyl ADT, suggesting that no apoptosis was triggered after 48 h of IGF-IR down-regulation, similarly to treatment with IGF-IR antisense oligonucleotides [22].

### Silencing of IGF-IR decreases *in vivo* tumor development

**Figure 4:**
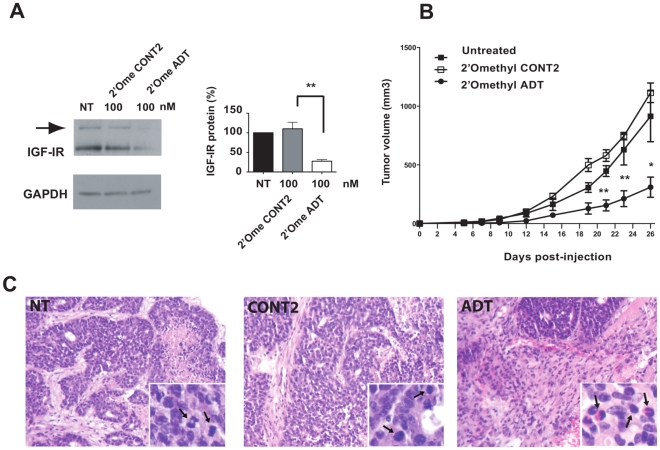
Transfection with siRNAs targeting IGF-IR alters *in vivo* growth of C4HD cells. (**A**) Western blot after 48 h transfection of C4HD cells with 2′-O-methyl siRNAs. Representative experiment is presented and quantitative analysis of three independent experiments is shown on the right of the blot with means ± SEM with black bars corresponding to mock transfected cells (NT). ** *P*<0.01. (**B**) After 48 h, treated cells were inoculated s.c. into mice. C4HD cells were transfected either with 2′-O-methyl ADT siRNA, which targets mIGF-IR (•), the control 2′-O-methyl CONT2 siRNA (□) or remained untreated (▪). Each data point represents the mean tumor volume ± SEM, n = 5. * *P*<0.05; ** *P*<0.01, ANOVA referred to untreated group. (**C**) Tissue sections of C4HD tumors obtained from mice in each group. Mice inoculated with untreated C4HD cells (NT), with C4HD cells transfected with the control 2′-O-methyl CONT2 siRNA (CONT2), or with the 2′-O-methyl ADT siRNA (ADT). Tumor shows atypical features, necrosis and several mitotic figures indicated with arrows (inset, H&E ×400). ADT sections show necrosis and inflammatory fibrosis (H&E ×40). Infiltration of lymphocytes and granulocytes is shown (inset, H&E ×400).

To explore the *in vivo* effect of IGF-IR silencing on breast cancer cell growth, we took advantage of the C4HD breast tumor model. We previously showed that IGF-IR plays a key role in *in vitro* and *in vivo* proliferation of the progestin-dependent C4HD tumor cells [21]. A functional autocrine loop involving IGF-I and IGF-IR participated in MPA-induced proliferation of C4HD cells. In this model, antisense phosphorothioates targeted to IGF-IR abolished the two main IGF-IR signaling pathways, AKT and MAPK [21]. Here, we transfected C4HD growing in 10 nM MPA with control or specific siRNAs targeting mIGF-IR. The silencing efficiency of mIGF-IR siRNAs in C4HD on IGF-IR ranged from 60 to 80% at 100 nM (Figure 4A). After 48 h, 2×10^6^ C4HD cells from each experimental group (n = 5) were inoculated s.c. into female BALB/c mice. Tumors in mice that had been given C4HD cells treated with IGF-IR siRNA (2′-O-methyl ADT) had significantly smaller mean tumor volumes and lower tumor growth rates compared with tumors from control groups (Figure 4B and Table S2). At day 26, a delay of 7 days in tumor growth was observed in groups injected with 2′-O-methyl ADT siRNA-transfected C4HD with respect to tumors that developed in mice injected with C4HD, and of 10 days with respect to tumors growing in mice injected with 2′-O-methyl CONT2 siRNA-transfected cells. Histopathological analysis was performed by H&E staining of histological sections obtained from tumors excised at day 26. C4HD tumors grown in mice treated with MPA were ductal mammary carcinomas composed of solid pseudolobules of highly cohesive glandular cells that seldom showed tubular differentiation, separated by scanty fibroblastic stroma (Figure 4C). In this experiment, about 30% of tumor mass from tumors that developed from 2′-O-methyl ADT siRNA-transfected C4HD cells showed fibrosis as well as lymphocytes and polymorphonuclear neutrophils (PMN) (Figure 4C and Table S3). Moreover, tumors that developed from siRNA treated cells also showed a significantly lower number of mitotic events than did tumors from animals receiving untreated C4HD or 2′-O-methyl control siRNA-transfected C4HD cells.

### C4HD cells transfected with IGF-IR-siRNA trigger features of an immune response

**Figure 5:**
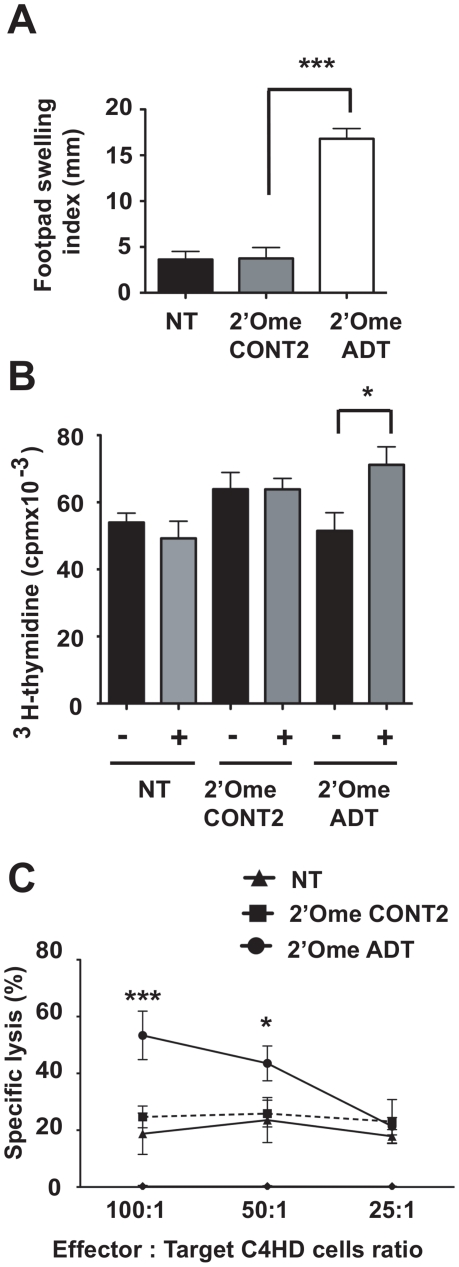
Immunization with C4HD cells treated with IGF-IR siRNAs. (**A**) Delayed-type hypersensitivity (DTH) response. BALB/c mice (n = 10) were immunized with three injections of irradiated C4HD cells (black bar) or irradiated 2′-O-methyl ADT siRNA (white bar) or 2′-O-methyl CONT2 siRNA (grey bar) transfected C4HD. NT, non-transfected cells. Data are presented as mean ± SEM; *** *P*<0.001. (**B**) Proliferation of splenocytes isolated from immunized mice. Incorporation of ^3^H thymidine was measured with isolated splenocytes co-cultured in the presence (+) or the absence (−) of C4HD cells preincubated with mitomycin C. Data are presented as mean ± SEM; * *P*<0.05. (**C**) Analysis of cytotoxic activity of splenocytes from immunized mice using a standard ^51^Cr release assay. Splenocytes (E) from mice immunized with 2′-O-methyl ADT (•) or 2′-O-methyl CONT2 (▪) transfected C4HD, or with irradiated C4HD cells (▴) were co-cultured with C4HD as target cells (T) at indicated E/T ratios. Mean ± SEM; * *P*<0.05; *** *P*<0.001.

C4HD cells growing in 10 nM MPA were transfected with 100 nM 2′-O-methyl ADT or 2′-O-methyl CONT2. After 48 h, cells were inactivated by irradiation and then used for immunization of mice according to previous procedures (Methods S1) [22]. A delayed-type hypersensitivity assay (DTH) was used to evaluate the immune response [22]. As shown in Figure 5A, DTH reactivity increased strongly in the group of mice immunized with 2′-O-methyl ADT-transfected C4HD compared with control groups, suggesting that a cellular immune response was triggered by IGF-IR down-regulation in C4HD. Animals were euthanized and the capacity of isolated splenocytes to proliferate *in vitro* in presence to C4HD cells was evaluated. Only splenocytes obtained from mice immunized with IGF-IR siRNA transfected C4HD significantly proliferated in response to mitomycin C-treated C4HD cells, whereas splenocytes from control groups did not respond (Figure 5B). To study the cytotoxic potential of the stimulated splenocytes isolated from mice immunized with siRNA-treated C4HD versus untreated C4HD cells, a ^51^Cr release assay was performed. Splenocytes prepared from mice injected with C4HD transfected with 2′-O-methyl ADT effectively induced lysis of C4HD cells, exhibiting the highest (∼50%) cytotoxic activity at an effector to target (E∶T) ratio of 100∶1 (Figure 5C). Lower cytotoxicity (20–30%) was observed over the range of E∶T ratios tested with the control groups. Based on results from DTH assays, splenocyte proliferation assays and cytotoxicity experiments, we conclude that the immunization protocol with ADT siRNA treated C4HD cells triggered some features of cellular immune response.

### Down-regulation of IGF-IR in breast cancer cells induces proinflammatory cytokines

**Figure 6:**
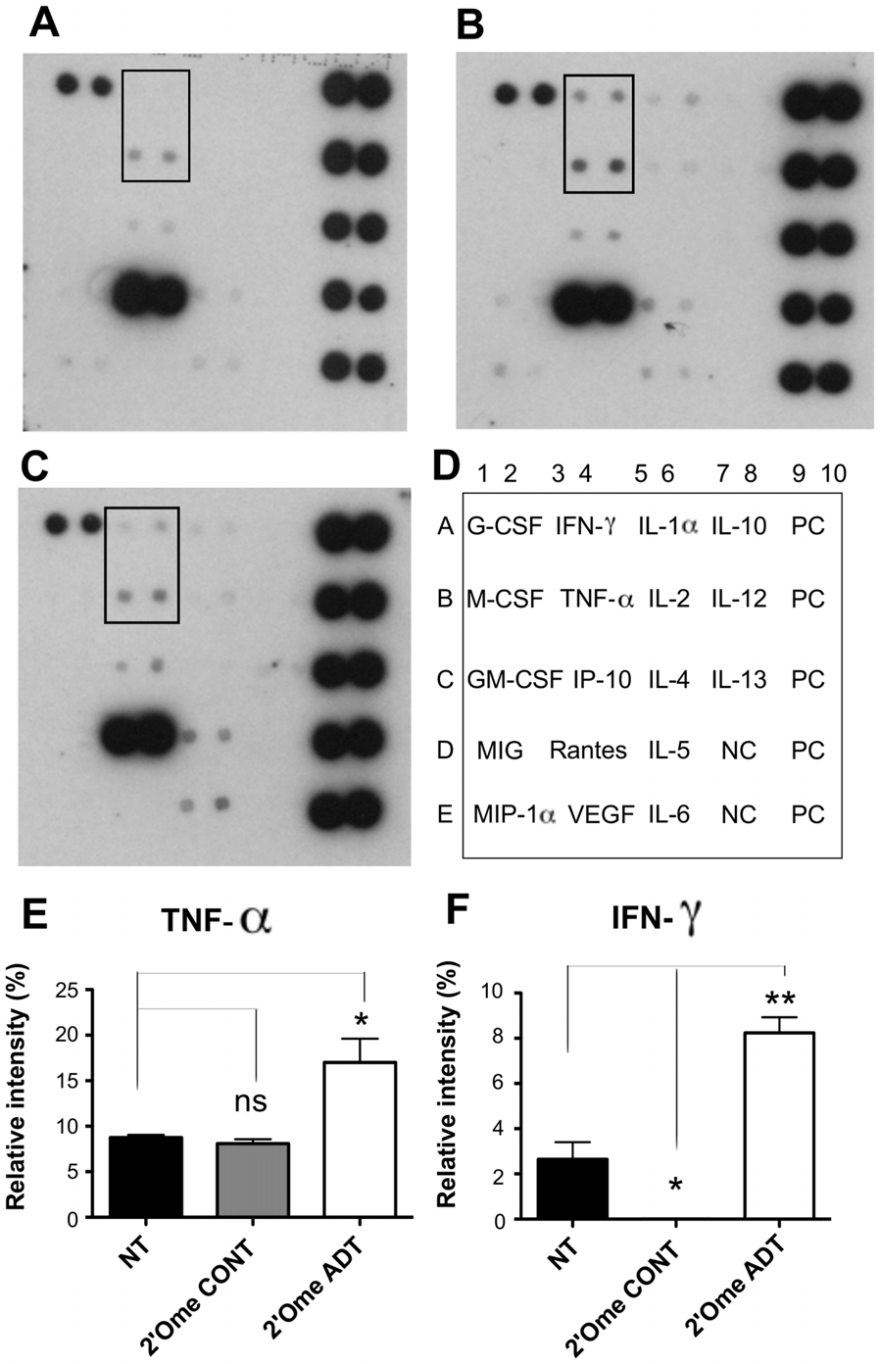
Profile of cytokine expression in C4HD cells treated with siRNAs targeting IGF-IR. Cytokine secretion in untreated C4HD cells (**C**) or C4HD cells transfected with 2′-O-methyl CONT2 (**A**), or 2′-O-methyl ADT (**B**) were analyzed 48 h post-transfection using a mouse cytokine antibody array (**D**). PC, array positive controls; NC, array negative controls. Graphs corresponded to densitometric analysis of membranes for TNF-α and IFN-γ expression (**E**, **F**). * *P*<0.05; ** *P*<0.01. Two independent cytokine antibody arrays were performed and gave similar results.

The effect of down-regulation of IGF-IR expression on cytokine production by C4HD breast cancer cells was profiled by using a cytokine antibody array. While most tested cytokines were not affected by IGF-IR downregulation, secretion of TNF-α and IFN-γ were stimulated significantly as demonstrated by enhanced signals in conditioned media of C4HD cells treated with IGF-IR siRNA as compared to untreated cells and 2′-O-methyl control siRNA transfected cells (Figure 6). Other cytokines such as RANTES, G-CSF and IL6 were not significantly affected by specific silencing of IGF-IR.

## Discussion

Small tyrosine kinase inhibitors and antibodies to IGF-IR aimed to block tumor growth *in vivo* are already tested in clinical trials. Inhibition of IGF-IR/IGF-I expression by nucleic acid based strategies could also be clinically useful as shown by a pilot study of *ex-vivo* treatment of malignant astrocytomas with antisense oligonucleotides targeted to IGF-IR [34]. This is especially stressed by the observation of IGF-I or IGF-IR inhibition with antisense based approaches leading to an antitumor host response [13], [17], [22], [23]. Other clinical trials have shown that hepatocarcinoma and glioblastoma cells treated with IGF-I antisense RNA constructs can serve as antitumor vaccines, inducing an effective immune response and a significant increase of median survival [35]. The triggering of immune host response after down-regulation of IGF-IR/IGF-I using nucleic acids was exemplified in three mouse syngenic models. Injection of melanoma cells pretreated with IGF-IR antisense oligonucleotide prevented the growth of s.c. injected untreated cells [18]. The presence of an immune response after down-regulation of IGF-IR using antisense RNA construct was also confirmed in a mouse neuroblastoma model [13]. Similar host response was described with antisense oligonucleotide treatment in a mouse breast cancer model [22]. However the mechanism linking the immune response and specific inhibition of IGF-I or IGF-IR expression has not yet be determined. It may occur *via* specific silencing of IGF-IR or *via* specific motifs present in nucleic acid sequences used to inhibit IGF-I or IGF-IR. Indeed, plasmids or oligonucleotides carrying non-methylated CpG sequences are known to trigger innate immune responses and can develop strong toxicity in human cells [36], [37]. Here, we used siRNAs rather than antisense RNA vectors or antisense oligonucleotides to analyze the effects of IGF-IR inhibition on immune response triggering. Since specific motifs in unmodified siRNA duplexes may activate cellular sensors of foreign RNA, leading to interferon induction and cell death [31], the siRNAs used in *in vivo* experiments were modified at uridine positions of the sense strand with 2′-O-methyl-uridine. This modification reportedly blocks the immunostimulatory effect of the RNA duplex without significantly attenuating RNAi [31]. However, we noticed a reduction of efficiency with modified siRNA even though 2′-O-methyl residues were not introduced at positions 9 and 10 in the sense strand; modifications at these positions are known to reduce RISC assembly [31]. Similarly, a detectable reduction in silencing efficacy was found with modified IGF-IR siRNAs, where 2′-O-methyl nucleotides were placed in alternating positions on both strands [38]. Moreover, we showed that our modified siRNAs targeting IGF-IR did not inhibit insulin receptor, which possess high homology with IGF-IR, unlike anti-IGF-IR TKI and antibody therapies that often induce inhibition of INS-R (Figure 2).

Inhibition of IGF-IR reduced AKT and ERK phosphorylation and consequently reduced the rate of cellular proliferation. Furthermore, down-regulation of IGF-IR arrested cells in the G0/G1 phase of the cell cycle; the major effect probably occurred at the G1-S interface and was presumably mediated through the PI3K-AKT and/or ERK pathways [1]. Interestingly, phosphorylation of AKT was inhibited at 25 nM ADT siRNA whereas no reduction of IGF-IR levels was observed. We proposed that at this concentration, IGF-IR was transiently inhibited leading to sustained decrease of p-AKT. After recovery of IGF-IR expression due to instability of siRNA and cell division, p-AKT was still strongly inhibited as previously observed by others [33], [39]. To achieve extended silencing of IGF-IR with strong inhibition of AKT phosphorylation, high concentration of siRNA was therefore chosen for *in vivo* experiments.

The inhibition of IGF-IR expression in C4HD mammary tumor cells significantly reduced tumor growth *in vivo*. This suppression of tumor growth might arise from several intracellular mechanisms and/or host antitumoral immune response as previously described [18]. The blockade of IGF-IR signaling may decrease either cell proliferation or increase apoptosis as shown with prostate cancer xenografts treated with IGF-IR antibody [40]. C4HD cell proliferation inhibition could occur *in vivo* while IGF-IR silencing decreased *in vitro* cell proliferation [41]. The absence of *in vitro* apoptosis after silencing does not preclude the possibility that prolonged IGF-IR silencing could induce a massive apoptosis *in vivo*. Indeed, it has been shown that antisense IGF-IR treatment can cause a partial growth arrest of glioblastoma without strong apoptosis and at the same time elicit almost complete cell death *in vivo* [42]. The reduced tumor growth could also result from activation of antitumoral immune host response, possibly induced by apoptotic cells [18], [43]. Histopathological analysis of tumors in mice treated with IGF-IR siRNA-transfected cells showed lower number of mitotic events concomitantly with lymphocytes and PMN infiltration, these cells being indicators of good prognosis in cancers. Moreover, we clearly demonstrated the presence of inflammatory features after vaccination with cells downregulated for IGF-IR according to our previous observations [22]. Our results confirmed that inhibition of IGF-IR might lead to immune response triggering through antisense RNA, antisense oligonucleotides and siRNAs [17], [22].

Tumors transfected with 2′-O-methyl ADT siRNA developed at later time points, suggesting loss of inhibitory siRNA during *in vivo* growth or emergence of tumor cells resistant to host immune response. Similarly, it was found that tumors may arise *in vivo* due to loss of the expression plasmid expressing IGF-IR antisense RNA [44]. We cannot exclude a recovery of IGF-IR after silencing depending on cell division rate. However, others showed that IGF-IR silencing with unmodified siRNAs can last for six days before re-expression [39]. To determine if arising tumors escape from immune response, previous tumor growth studies used immune deficient mice [13], [45]. Interestingly, growth of glioblastoma or neuroblastoma cells downregulated for IGF-IR in athymic nude mice was delayed, but to a lesser extent than in syngenic rodents. This delay in tumor growth of glioblastoma was found proportional to the extent of cell apoptosis induced by IGF-IR antisense treatment [45], [46]. Nevertheless, it was shown that antitumoral immune host response triggered by IGF-IR downregulation in syngenic animals was highly effective when most of the injected cells underwent massive apoptosis [43]. It was therefore proposed that expression of antisense IGF-IR induced apoptosis concomitantly with the secretion of cytotoxic substances, both potentially stimulating immune response [17], [43]. Similar mechanism may occur with C4HD cells in syngenic mice, despite the absence of *in vitro* apoptosis induced by IGF-IR silencing.

Our study provides the first demonstration that inhibition of IGF-IR using 2′-O-methyl siRNA increased secretion of TNF-α and IFN-γ, two proinflammatory cytokines. These two cytokines are multifunctional and are produced mainly by activated macrophages and lymphocytes, although non-hematopoietic cells such as malignant cells or tumor stroma cells also synthesize TNF-α [47]. This latter is a key mediator of the inflammatory response, and can play a dual role in tumor environment, inducing paradoxical effects. Due to its proapoptotic activity, TNF-α can inhibit *in vitro* growth of some tumor cells including breast tumor cell lines [48]. Moreover, forced expression of LIGHT, a TNF superfamily member, in tumor tissue induced priming of naive T cells and led to rejection of established tumors in mice [49]. Besides TNF-α action to suppress proliferation and induce apoptosis in a variety of cancer cells, it can also exert a growth-promoting effect on normal epithelia [50]. Its production by malignant or host cells in tumor microenvironment was associated with increased malignancy of tumors and favored metastasis [51], [52], [53]. We have also shown that exogenous supply of TNF-α to C4HD cells promoted their *in vitro* growth through NF-κB dependant pathways [29]. Consequently this cytokine is able to exert pleiotropic effects on cells, with the paradox outcome of cell death or growth, depending on the context [54]. TNF-α may also act concomitantly with IFN-γ to block tumor stroma formation [55]. Both cytokines can sensitize metastatic colon carcinoma cells to TRAIL-induced apoptosis *in vitro* [56]. They play a role as immunoadjuvant through induction of MHC class I molecules, activate immature dendritic cells or trigger an adaptative immune response through induction of CD8^+^ T cells [57], [58].

Our data support the existence of crosstalk between IGF-IR axis, immune response and secretion of TNF-α and IFN-γ cytokines. Interactions between endocrine and immune systems are well documented [59], [60]. Indeed, IGF-I is known to play a prominent role in the regulation of immunity and inflammation [61]. Anti apoptotic IGF-I can reduce TNF-α cytotoxicity in the inflammatory response to acute renal injury [62], whereas it may also potentiate TNF-α induced apoptosis in specific cell types [63]. Proinflammatory cytokines often acted as negative regulatory signals that temper the action of hormones and growth factors [59]. TNF-α blocked growth of breast cancer cells by impairing IGF-IR signaling [64]. TNF-α also promoted neurodegeneration through inhibition of IGF-I survival signal [65]. Interestingly, TNF-α and IFN-γ were shown to affect IGF-IR promoter activity and decrease IGF-IR protein levels in human sarcoma cell lines [66]. Whereas high expression of TNF-α was therefore correlated with diminished IGF-IR levels, our work showed that silencing IGF-IR in the C4HD breast cancer model increased TNF-α and IFN-γ secretion. We may speculate that when IGF-IR is silenced, TNF-α and IFN-γ secretion contributed to decreased tumor growth either priming T cell response, blocking tumor stroma or triggering tumor apoptosis. Also, we cannot exclude that these cytokines elicit an anti apoptotic effect to counteract the tumor inhibition induced by IGF-IR silencing. Moreover, in addition to these cytokines, triggering of host cell response would need involvement of multiple factors, that warrant further investigations such as genome-wide expression profiling or complementary cytokine antibody array studies [67], [68].

Presently, siRNA-based therapies are being evaluated clinically. However, delivery of these large and highly charged molecules still represents a major barrier to therapeutic application. Novel RNAi delivery methods are therefore under intense investigation [69]. The association of efficient delivery vehicles and siRNA sequences is essential for achieving specific and efficient antitumor immune response. For example, modifications of siRNAs by introducing micro-RNA motifs, aptamers or polymers could be envisaged to obtain bifunctional molecules leading to specific silencing activities associated with proinflammatory properties, cell targeting or cell penetration increase [70], [71], [72]. Moreover, vehicles like polyethyleneimine may also harbor intrinsic immunostimulatory activities, which in association with siRNA may enhance antigen-presenting capacity of mouse tumor-associated dendritic cells and induce direct tumoricidal activity [73].

Here we showed that 2′-O-methyl modified siRNA targeted to mouse IGF-IR are able to block IGF-IR signaling and tumor growth, by inducing features of antitumor immune response. In further studies, siRNAs targeting IGF-IR will be modified and complexed to specific carriers with adjuvant properties, to improve the effect of IGF-IR downregulation and consequently modulate antitumor immune responses with the goal of developing local and systemic RNAi therapy. It remains to be assessed whether TNF-α and IFN-γ are effective biomarkers for the efficacy of anti-IGF-IR therapy.

## Supporting Information

Methods S1 **Supplementary methods.**
(DOC)

Figure S1 **Alignment of mouse IGF-IR and INS-R mRNAs.** Clustal 2.0.12 multiple alignments of mIGF-IR (Genbank NM_010513) and mouse insulin receptor (Genbank NM_010568) were performed using the EMBL-EBI web site. Start positions refer to the translation initiation codons. For clarity, only regions with siRNA binding sites are shown and these binding sites are highlighted in gray. The LKD siRNA sequence is underlined. There is a global similarity of 0.62 between mIGF-IR and mINS-R: similarity in siRNA sites are indicated in percentages (Id).
(PDF)

Table S1 **siRNA sequences.** ^1^siRNA are targeted to IGF-IR in three species, M, mouse; R, rat; H, human. ^2^Positions on the target gene were indicated from the mature protein, without signal peptide. ^3^Underlined bold u indicates 2′-O-methyluridine residue. ^4^References 1. Niu, J, Xu, Z, Li, X-N, Han, Z. (2007) siRNA-mediated type 1 insulin-like growth factor receptor silencing induces chemosensitization of a human liver cancer cell line with mutant P53. Cell Biology International 31: 156–164. 2. Chalk, AM, Wahlestedt, C, Sonnhammer, ELL. (2004) Improved and automated prediction of effective siRNA. Biochem Biophys Res Commun 319: 264–274. 3. Da Silva Xavier, G, Qian, Q, Cullen, PJ, Rutter, GA. (2004) Distinct roles for insulin and insulin-like growth factor-1 receptors in pancreatic beta-cell glucose sensing revealed by RNA silencing. Biochem J 377: 149–158. 4. Naito, Y, Yamada, T, Ui-Tei, K, Morishita, S, Saigo, K. (2004) siDirect: highly effective, target-specific siRNA design software for mammalian RNA interference. Nucleic Acids Res 32: W124-W129. 5. Yeh, AH, Bohula, EA, Macaulay, VM. (2006) Human melanoma cells expressing V600E B-RAF are susceptible to IGF1R targeting by small interfering RNAs. Oncogene 25: 6574–6581. 6. Rosengren, L, Vasilcanu, D, Vasilcanu, R, Fickenscher, S, Sehat, B, et al. (2006) IGF-1R tyrosine kinase expression and dependency in clones of IGF-1R knockout cells (R-). Biochem Biophys Res Commun 347: 1059–1066. 7. Carboni, JM, Lee, AV, Hadsell, DL, Rowley, BR, Lee, FY, et al. (2005) Tumor development by transgenic expression of a constitutively active insulin-like growth factor I receptor. Cancer Research 65: 3781–3787. 8. Rochester, MA, Riedemann, J, Hellawell, GO, Brewster, SF, Macaulay, VM. (2005) Silencing of the IGF1R gene enhances sensitivity to DNA-damaging agents in both PTEN wild-type and mutant human prostate cancer. Cancer Gene Ther 12: 90–100. MTN, LDA and DIN siRNAs were designed using online software described in reference 2 and 4.
(PDF)

Table S2 **Tumor growth rates in mice injected with siRNA-transfected C4HD cells.** ^1^Mice treated with a 40 mg s.c. MPA depot were inoculated in the flank opposite with C4HD cells transfected with 2′-O-methyl siRNA targeting IGF-IR (ADT) or with a control 2′-O-methyl siRNA (CONT2) or with untreated C4HD cells. At day 26, tumor volume and percentage of growth inhibition in tumors from mice injected with ADT siRNA transfected cells were compared to those of mice injected with control siRNA or with untreated C4HD cells. ^2^Tumor volume in mm^3^ ± SEM, n = 5. ^3^Growth rate are expressed in mm^3^/day ± SEM, n = 5. § vs &, P<0.05; § vs £, P<0.01.
(PDF)

Table S3 **Characteristics of tumors obtained from mice injected with C4HD cells transfected with 2′-O-methyl siRNAs.** ^1^Number of mitotic bodies per high power field (HPF). ^2^GM1-2 indicates 0-5 mitosis per 10 HPF; GM3 indicates >10 mitoses per 10 HPF. ^3^Polymorphonuclear neutrophils.
(PDF)
