# Supplementary material for: Small Interfering RNA Targeted to IGF-IR Delays Tumor Growth and Induces Proinflammatory Cytokines in a Mouse Breast Cancer Model
Source: PLoS One. 2012 Jan 3;7(1):e29213. doi: 10.1371/journal.pone.0029213 (PMC3250415; doi:10.1371/journal.pone.0029213)
Supplement: Methods S1 — Supplementary methods. (DOC) [file pone.0029213.s001.doc]

**Supplementary Materials and methods**

**siRNA synthesis and siRNA nomenclature.** siRNA (sequences in Table S1) were synthesized on an ASM-800 synthesizer (Biosset, Russia) at 0.4 μmol scale using optimized standard solid phase phosphoramidite synthesis [1]. Reagents for the synthesis and deprotection were obtained from Panreac (Spain) and Sigma-Aldrich (USA). 5′-O-Dimethoxytrityl-2′-O-tert-butyldimethylsilyl-N-acyl-ribonucleoside-3′-phosphoramidites, 5′-O-dimethoxytrityl-2′-O-methyluridine-3′-phosphoramidite, 5′-O-dimethoxytrityl-2'-deoxythymidine-3′-phosphoramidite and 5′-O-dimethoxytrityl-2'-deoxythymidine-3′-lcaa-CPG were purchased from Glen Research (USA). 5-ethylthiotetrazole (0.25 M) has been used as activator with 4, 6 or 10 min coupling step for thymidine-3′-phosphoramidite, 2′-O-methyluridine-3′-phosphoramidite and 2′-O-tert-butyldimethylsilylribonucleoside-3′-phosphoramidites, respectively. Upon completion of the synthesis, oligonucleotides were cleaved from the support and deprotected in 40 % methylamine in water at 65°C for 15 min. 2′-O-Silyl groups were removed by treatment with a mixture of triethylamine trihydrofluoride/N-methyl-2-pyrrolidinone/triethylamine at 65°C for 1.5 hours. Deprotected oligonucleotides were isolated by denaturing 15 % PAGE, visualized by UV-shadowing, extracted from the gel by the crush and soak method with 0.3 M NaClO_4_ and desalted on Sep-Pak C18 cartridge (Waters, USA) followed by precipitation as sodium salts. The purified oligonucleotides were characterized by 15% PAGE and by MALDI-TOF mass spectra on a Reflex III (Bruker Daltonics, Germany). Some siRNA sequences were purchased from Eurogentec (Seraing, Belgium). The 3’ end of siRNA strands had two deoxythymidines for nuclease protection. Certain siRNAs were modified with 2’ O-methyl nucleotides. During automatic synthesis we used 2'-O-methyluridine phosphoramidite instead of uridine (Table S1). Sense and antisense strands were annealed in sterile RNase-free siRNA buffer (50 mM Tris, pH 7.5, 100 mM NaCl) and stored in aliquots at -70°C. Concentrations of all siRNAs and primers were determined spectrophotometrically at 25°C with molar extinction coefficients calculated as previously described [2]. The three letters in the name of each siRNA correspond to the first three amino acids of the targeted protein coded by the first 9 nucleotides of sense strand of the corresponding target. Positions on the target gene are indicated based on the mature protein without signal peptide. All of siRNA sequences were checked for uniqueness by BLAST search against mouse sequences in the ENSEMBL database. Two siRNA duplexes that do not target any known mammalian genes were used as negative controls [3,4]. The hRluc siRNA, which is specific for the synthetic Renilla luciferase gene was designed using siMAX design tool and synthesized by Eurofins MWG Operon, Ebersberg, Germany.

**Design of siRNAs targeted to mouse IGF-IR.** To identify siRNAs that are potent and sequence-specific inhibitors of mouse IGF-IR, several siRNAs were designed using either literature guidelines or siRNA design web softwares [5,6]. Human IGF-IR was targeted previously with siRNA [7]. The siRNA designed against α subunit of mouse IGF-IR used in pancreatic islet B cells had one mismatch at position 16 of sense strand as compared to mouse IGF-IR sequence present in GenBank and our LKD siRNA [8]. Another sequence targeting the ß subunit of human IGF-IR efficiently down-regulated mouse IGF-IR in mouse embryonic cells, despite the presence of 2 mismatches to the corresponding sequences of murine IGF-IR DNA [9]. We therefore chose the complementary sequence corresponding to this ß-subunit region of mouse IGF-IR; this siRNA is referred to as ADT siRNA. The CMV siRNA corresponds to an siRNA targeted to human IGF-IR with two changes at the 5’end of sense strand to recognize the mouse sequence [10]. Other sequences are based on previous publications [4,11].

**RNA preparation and quantitative RT-PCR**

Total RNA isolation from EMT6 cells transfected with siRNAs was performed using RNeasy Mini kits (Qiagen, Courtaboeuf, France). Total RNA (0.5 µg), checked by absorbance spectroscopy using a Nanodrop (Thermo Fisher Scientific), was reverse-transcribed to generate first strand cDNA using random nonamers and AffinityScript enzyme (Stratagene, Agilent Technologies, Massy, France). Quantitative RT-PCR was performed on an Mx3000P system (Stratagene) using Brilliant SYBR Green QPCR Master Mix (Stratagene) and two primers designed with Universal ProbeLibrary Assay web Design Center (Roche Applied Science). Primer sequences chosen for intron-spanning assays were for mouse IGF-IR (GenBank Accession #: NM_010513; forward, 5’-GAGAATTTCCTTCACAATTCCATC-3’; reverse, 5’-CCTCTCTGCAGTACGTTCAC-3’). The mouse ß glucuronidase (Gusb) was used as internal loading control since its expression did not change over a 72 h period with the addition of siRNAs (NM_010368; forward, 5’-GAGGATCAACAGTGCCCATT-3’; reverse, 5’-TGGAGGGGAAACTCCGAC-3’). The PCR conditions were as follows: 15 min at 94°C, 40 cycles of 30 s at 94°C, 30 s at 55°C, and 30 s at 72°C. To determine relative quantities of mRNA, the relative standard curve method was used from standard curves generated with serially diluted solutions of cDNA prepared from untreated EMT6 cells. PCR efficiencies with all primer sets were in a range between 95 and 105%. All PCR assays were conducted in duplicate for measurement of each sample. Values from two independent transfection experiments were averaged.

**Gel electrophoresis, immunoblotting and quantification.** Proteins from EMT6 transfected cells were analyzed by Western blot using cell lysates prepared in RIPA buffer (0.05 M Tris-HCl, pH 7.4, 0.15 M NaCl, 0.25% deoxycholic acid, 1% NP-40, 1 mM EDTA) containing 1 mM sodium orthovanadate, 25 mM NaF and protease inhibitor cocktail (Roche Applied Biosciences). Protein lysates from C4HD tumors growing in mice subjected to different treatments were prepared as previously described [12,13]. C4HD proteins were solubilized in sample buffer (60 mM Tris-HCl, pH 6.8, 2% SDS, 10% glycerol and 0.01% bromophenol blue) and subjected to SDS-PAGE. After determination of concentration using a Bio-Rad Protein Assay, protein samples were separated by 8% SDS-PAGE and then electro-transferred onto a polyvinylidene difluoride membrane or nitrocellulose (C4HD). The membranes were immunoblotted using various primary antibodies (anti-IGF-IRß diluted 1:200; anti-INSR, 1:200; anti-AKT, 1:1,000; anti-ERK1/2, 1:1,000; anti ß-actin, 1:5,000; anti GAPDH, 1:5,000) in the blocking solution at 4°C overnight followed by the appropriate horseradish peroxidase-conjugated secondary antibody (at room temperature for 1 h) and then developed by the chemiluminescence method on Kodak X-OMAT film. Band densitometry was performed using NIH ImageJ 1.43 software following the method outlined at this web site [14]. Immunoblots shown are representative of at least two experiments. Quantitative analysis of these independent assays are shown next one example of Western blot. For mouse cytokine-antibody arrays, quantification was done similarly, with normalization to the total signal observed on membranes. Variability between positive controls was found identical. Signals of duplicates were compared between membranes obtained with supernatant of C4HD cells transfected by siRNAs. ANOVA was used to determine significant difference of cytokine expression. Two independent cytokine antibody arrays were performed and gave similar results.

**Immunization of mice with transfectants.** **delayed-type hypersensitivity, splenocyte proliferation and cytotoxic assays.** For immunization of mice, C4HD cells were transfected for 48 h with 100 nM 2’-O-methyl ADT siRNA or the 2-’O-methyl CONT2 siRNA or mock transfected with Dharmafect I. Cells were then inactivated by irradiation with 50 Gy of ^60^Co. BALB/c mice were injected s.c. three times at two-week intervals in the left flank with 2x10^6^ cells. For the delayed-type hypersensitivity (DTH) assay, mice were immunized as described above, and one week after the last immunization were challenged s.c. with 2x10^5^ irradiated C4HD untreated cells in the left footpad and with PBS in the right footpad. The DTH response was determined 48 h later by measuring footpad swelling with a dial caliper. Results were expressed as footpad swelling index obtained by subtracting the right footpad thickness (baseline) from that of the left (experimental). Ten mice were included in each experimental group. For the splenocyte proliferation assay, mice were immunized as described above, and two weeks after the last injection splenocytes were isolated from four sacrificed animals and cultured in flat-bottom 96-well plates for 5 days at 2x10^6^ cell/ml in the presence or the absence of 4x10^4^ C4HD cells per ml that had previously been incubated with 50 µg/ml mitomycin C (Sigma-Aldrich) at 37°C for 1 h. The assay was performed as previously described using a pulse with 0.5 µCi of [^3^H] thymidine for 16 h [15]. Cells were then harvested and incorporation of [^3^H] thymidine was used as a measure of DNA synthesis. Measurements were performed in quadruplicate. Cytotoxicity was determined using a standard *in vitro* ^51^Cr release assay measuring lysis of C4HD cells (target cells) by mouse splenocytes (effector cells), which were obtained from mice subjected to the immunization protocols described. Splenocytes were co-cultured for 5 days with mitomycin-treated C4HD cells. C4HD cells were first labeled with 100 µCi of ^51^Cr (Na_2_^51^CrO_4_; specific activity, 0.5 Ci/mg; NEN DuPont) for 1 h at 37°C and were washed four times. Cells were then plated at 1x10^4^ cells/well in a round-bottom 96-well plate in E:T ratios of 100, 50 and 25 and were incubated for 14 h at 37°C. After incubation, radioactivity released was measured in a gamma counter. Wells containing only target cells in the absence or presence of 1% Triton X-100 determined spontaneous and maximal ^51^Cr release, respectively. Percentage of specific target cell lysis was calculated using ((E – S)/(T – S)) x 100, where E is the average experimental release, S the average spontaneous release, and T the average total release.

**Supplementary References**
