# Supplementary material for: Small Interfering RNA Targeted to IGF-IR Delays Tumor Growth and Induces Proinflammatory Cytokines in a Mouse Breast Cancer Model
Source: PLoS One. 2012 Jan 3;7(1):e29213. doi: 10.1371/journal.pone.0029213 (PMC3250415; doi:10.1371/journal.pone.0029213)
Supplement: Figure S1 — Alignment of mouse IGF-IR and INS-R mRNAs. Clustal 2.0.12 multiple alignments of mIGF-IR (Genbank NM_010513) and mouse insulin receptor (Genbank NM_010568) were performed using the EMBL-EBI web site. Start positions refer to the translation initiation codons. For clarity, only regions with siRNA binding sites are shown and these binding sites are highlighted in gray. The LKD siRNA sequence is underlined. There is a global similarity of 0.62 between mIGF-IR and mINS-R: similarity in siRNA sites are indicated in percentages (Id). (PDF) [file pone.0029213.s002.pdf]

```
                                                                            Pos   Name      Id
mIGF-IR       ATGAAGTCTGGCTCCGGAGGAGGGTCCCCGACCTCGC---TGTGGGGGCTCGTGTTTCTC 57    KSG       0.42
mINS-R        ATGGGCTTCGGGAGAGGATGTGAGACGACGGCTGTGCCATTGCTGGTGGCCGTGGCCGCG 60
              ***   *  **    *** * * * *  ** *   **   **  ** *  ****

mIGF-IR     //ATCCGCAACGACTATCAGCAGCTGAAGCGCCTGGAAAACTGCACGGTGATCGAGGGCTTC 174   DYQ       0.37
mINS-R      //ATCCGGAACAACCTGACCAGGCTACATGAGCTGGAGAACTGCTCAGTCATTGAGGGCCAT 177
              ***** *** **        ***  *    ***** ****** * ** ** ******

mIGF-IR     //CTGGTCATCTTCGAGATGACCAATCTCAAGGATATTGGGCTTTATAATCTGAGGAACATT 408   MTN/LKD
mINS-R      //CTGGTTATCTTCGAGATGGTCCACCTGAAGGAGCTGGGGCTTTATAACCTCATGAACATC 417     0.68/0.73
              ***** ************  * * ** *****  * *********** ** * ******

mIGF-IR     //TGGTCTCTCATCTTGGATGCGGTGTCCAATAACTACATTGTGGGGAACAA-GCCCCCGA- 526   LDA       0.69
mINS-R      //TGGTCCCGTATCCTGGATTCTGTGGAGGACAACTACATTGTACTGAACAAAGATGACAAC 537
              ***** *  *** ***** * ***    * ***********   ****** *    * *

mIGF-IR     //ACCATCAACAATGAGTACAACTACCGCTGCTGGACCACAAATCGCTGCCAGAAAATGTGC 645   NNE       0.31
mINS-R      //GTCATCAATGGGCAGTTTGTGGAACGGTGCTGGACACACAGTCATTGTCAGAAAGTTTGC 657
                ******     ***      * ** ********    * **  ** ****** * ***

mIGF-IR     //CGCCAGAGCAAAGGGGACATAAACACCAGGAACAACGGAGAGCGAGCTTCCTGTGAAAGT 1473  DIN       0.58
mINS-R      //CGTCAGGAGAGGAACGACATTGCCCTGAAGACCAATGGGGACCAGGCATCGTGTGAAAAT 1491
              ** ***   *     *****   *   * ** *** ** ** *  ** ** ******* *

mIGF-IR     //CCAACACGACCATGTCCAGCCGAAGCAGGAACACCACGGTAGCTGACACCTACAATATCA 2299  ADT       0.52
mINS-R      //GGAATGTGACAG---CCACCACACTCACACTTCCAGATTTCCCCAACGTCTCCTCTACCA 2383
                **   ***     *** *  *  **      *     *  *  **  ** *  ** **

mIGF-IR     //CAGAGACCTTGCTGCTAGGAACTGCATGGTAGCCGAAGATTTCACAGTCAAAATTGGAGA 3464  CMV       0.84
mINS-R      //CCGGGACCTGGCAGCTCGAAACTGCATGGTTGCCCATGATTTTACTGTCAAAATTGGAGA 3500
              * * ***** ** *** * *********** *** * ***** ** **************
```

Figure S1. Alignment of mouse IGF-IR and INS-R mRNAs.

Clustal 2.0.12 multiple alignments of mIGF-IR (Genbank NM_010513) and mouse insulin receptor (Genbank NM_010568) were performed using the EMBL-EBI web site. Start positions refer to the translation initiation codons. For clarity, only regions with siRNA binding sites are shown and these binding sites are highlighted in gray. The LKD siRNA sequence is underlined. There is a global similarity of 0.62 between mIGF-IR and mINS-R: similarity in siRNA sites are indicated in percentages (Id).
