# Supplementary material for: Small Interfering RNA Targeted to IGF-IR Delays Tumor Growth and Induces Proinflammatory Cytokines in a Mouse Breast Cancer Model
Source: PLoS One. 2012 Jan 3;7(1):e29213. doi: 10.1371/journal.pone.0029213 (PMC3250415; doi:10.1371/journal.pone.0029213)
Supplement: Table S1 — siRNA sequences. 1siRNA are targeted to IGF-IR in three species, M, mouse; R, rat; H, human. 2Positions on the target gene were indicated from the mature protein, without signal peptide. 3Underlined bold u indicates 2′-O-methyluridine residue. 4References 1. Niu, J, Xu, Z, Li, X-N, Han, Z. (2007) siRNA-mediated type 1 insulin-like growth factor receptor silencing induces chemosensitization of a human liver cancer cell line with mutant P53. Cell Biology International 31: 156–164. 2. Chalk, AM, Wahlestedt, C, Sonnhammer, ELL. (2004) Improved and automated prediction of effective siRNA. Biochem Biophys Res Commun 319: 264–274. 3. Da Silva Xavier, G, Qian, Q, Cullen, PJ, Rutter, GA. (2004) Distinct roles for insulin and insulin-like growth factor-1 receptors in pancreatic beta-cell glucose sensing revealed by RNA silencing. Biochem J 377: 149–158. 4. Naito, Y, Yamada, T, Ui-Tei, K, Morishita, S, Saigo, K. (2004) siDirect: highly effective, target-specific siRNA design software for mammalian RNA interference. Nucleic Acids Res 32: W124-W129. 5. Yeh, AH, Bohula, EA, Macaulay, VM. (2006) Human melanoma cells expressing V600E B-RAF are susceptible to IGF1R targeting by small interfering RNAs. Oncogene 25: 6574–6581. 6. Rosengren, L, Vasilcanu, D, Vasilcanu, R, Fickenscher, S, Sehat, B, et al. (2006) IGF-1R tyrosine kinase expression and dependency in clones of IGF-1R knockout cells (R-). Biochem Biophys Res Commun 347: 1059–1066. 7. Carboni, JM, Lee, AV, Hadsell, DL, Rowley, BR, Lee, FY, et al. (2005) Tumor development by transgenic expression of a constitutively active insulin-like growth factor I receptor. Cancer Research 65: 3781–3787. 8. Rochester, MA, Riedemann, J, Hellawell, GO, Brewster, SF, Macaulay, VM. (2005) Silencing of the IGF1R gene enhances sensitivity to DNA-damaging agents in both PTEN wild-type and mutant human prostate cancer. Cancer Gene Ther 12: 90–100. MTN, LDA and DIN siRNAs were designed using online software described in reference 2 and 4. (PDF) [file pone.0029213.s003.pdf]

Table S1. siRNA sequences.

Name	Target[1]	Position[2]	Size (bp)	Strand	Sequence[3]	References[4]
KSG	IGF-IR(M+H+R)	3	21	S	5'-GAAGUCUGGCUCCGGAGGAdTdT-3'	-
				AS	3'-dTdTCUUCAGACCGAGGCCUCCU-5'	
DYQ	IGF-IR(M+H+R)	123	21	S	5'-CGACUAUCAGCAGCUGAAGdTdT-3'	[1]
				AS	3'-dTdTGCUGAUAGUCGUCGACUUC-5'	
MTN	IGF-IR(M+H+R)	351	21	S	5'-GAUGACCAAUCUCAAGGAUdTdT-3'	[2]
				AS	3'-dTdTCUACUGGUUAGAGUUCCUA-5'	
LKD	IGF-IR(M+H+R)	363	21	S	5'-UCUCAAGGAUAUUGGGCUUdTdT-3'	[3]
				AS	3'-dTdTAGAGUUCCUAUAACCCGAA-5'	
LDA	IGF-IR(M+H+R)	482	25	S	5'-UGGAUGCGGUGUCCAAUAACUACdTdT-3'	[4]
				AS	3'-dTdTACCUACGCCACAGGUUAUUGAUG-5'	
NNE	IGF-IR(M+H+R)	594	21	S	5'-CAAUGAGUACAACUACCGCdTdT-3'	[5]
				AS	3'-dTdTGUUACUCAUGUUGAUGGCG-5'	
DIN	IGF-IR(M+H+R)	1429	21	S	5'-GACAUAAACACCAGGAACAdTdT-3'	[2]
				AS	3'-dTdTCUGUAUUUGUGGUCCUUGU-5'	
ADT	IGF-IR(M+R)	2281	21	S	5'-GCUGACACCUACAAUAUCAdTdT-3'	[6]
				AS	3'-dTdTCGACUGUGGAUGUUAUAGU-5'	
CMV	IGF-IR(M)	3426	21	S	5'-CUGCAUGGUAGCCGAAGAUdTdT-3'	[7]
				AS	3'-dTdTGACGUACCAUCGGCUUCUA-5'	
2'Ome ADT	IGF-IR(M+R)	2281	21	S	5'-GC**u**GACACCUACAAUA**u**CAdTdT-3'	-
				AS	3'-dTdTCGACUGUGGAUGUUAUAGU-5'	
CONT1	none	-	21	S	5'-GUCACACCGAUAAGUCACAdTdT-3'	[8]
				AS	3'-dTdTCAGUGUGGCUAUUCAGUGU-5'	
CONT2	none	-	21	S	5'-UUCUCCGAACGUGUCACGUdTdT-3'	[5]
				AS	3'-dTdTAAGAGGCUUGCACAGUGCA-5'	
2'Ome CONT1	none	-	21	S	5'-G**u**CACACCGAUAAG**u**CACAdTdT-3'	-
				AS	3'-dTdTCAGUGUGGCUAUUCAGUGU-5'	
2'Ome CONT2	none	-	21	S	5'-UUCUCCGAACG**u**G**u**CACGUdTdT-3'	-
				AS	3'-dTdTAAGAGGCUUGCACAGUGCA-5'	
hRluc	Renilla luciferase	105	21	S	5'-CUACUAUGAUUCCGAGAAGdTdT-3'	-
				AS	3'-dTdTGAUGAUACUAAGGCUCUUC-5'	

[1]siRNA are targeted to IGF-IR in three species, M, mouse; R, rat; H, human. [2]Positions on the target gene were indicated from the mature protein, without signal peptide. [3]Underlined bold u indicates 2'-O-methyluridine residue. [4]References 1. Niu, J, Xu, Z, Li, X-N, Han, Z. (2007) siRNA-mediated type 1 insulin-like growth factor receptor silencing induces chemosensitization of a human liver cancer cell line with mutant P53. Cell Biology International 31: 156-164. 2. Chalk, AM, Wahlestedt, C, Sonnhammer, ELL. (2004) Improved and automated prediction of effective siRNA. Biochem Biophys Res Commun 319: 264-274. 3. Da Silva Xavier, G, Qian, Q, Cullen, PJ, Rutter, GA. (2004) Distinct roles for insulin and insulin-like growth factor-1 receptors in pancreatic beta-cell glucose sensing revealed by RNA silencing. Biochem J 377: 149-158. 4. Naito, Y, Yamada, T, Ui-Tei, K, Morishita, S, Saigo, K. (2004) siDirect: highly effective, target-specific siRNA design software for mammalian RNA interference. Nucleic Acids Res 32: W124-W129. 5. Yeh, AH, Bohula, EA, Macaulay, VM. (2006) Human melanoma cells expressing V600E B-RAF are susceptible to IGF1R targeting by small interfering RNAs. Oncogene 25: 6574-6581. 6. Rosengren, L, Vasilcanu, D, Vasilcanu, R, Fickenscher, S, Sehat, B, et al. (2006) IGF-1R tyrosine kinase expression and dependency in clones of IGF-1R knockout cells (R-). Biochem Biophys Res Commun 347: 1059-1066. 7. Carboni, JM, Lee, AV, Hadsell, DL, Rowley, BR, Lee, FY, et al. (2005) Tumor development by transgenic expression of a constitutively active insulin-like growth factor I receptor. Cancer Research 65: 3781-3787. 8. Rochester, MA, Riedemann, J, Hellawell, GO, Brewster, SF, Macaulay, VM. (2005) Silencing of the IGF1R gene enhances sensitivity to DNA-damaging agents in both PTEN wild-type and mutant human prostate cancer. Cancer Gene Ther 12: 90-100. MTN, LDA and DIN siRNAs were designed using online software described in reference 2 and 4.
