# Supplementary material for: Small Interfering RNA Targeted to IGF-IR Delays Tumor Growth and Induces Proinflammatory Cytokines in a Mouse Breast Cancer Model
Source: PLoS One. 2012 Jan 3;7(1):e29213. doi: 10.1371/journal.pone.0029213 (PMC3250415; doi:10.1371/journal.pone.0029213)
Supplement: Table S2 — Tumor growth rates in mice injected with siRNA-transfected C4HD cells. 1Mice treated with a 40 mg s.c. MPA depot were inoculated in the flank opposite with C4HD cells transfected with 2′-O-methyl siRNA targeting IGF-IR (ADT) or with a control 2′-O-methyl siRNA (CONT2) or with untreated C4HD cells. At day 26, tumor volume and percentage of growth inhibition in tumors from mice injected with ADT siRNA transfected cells were compared to those of mice injected with control siRNA or with untreated C4HD cells. 2Tumor volume in mm3 ± SEM, n = 5. 3Growth rate are expressed in mm3/day ± SEM, n = 5. § vs &, P<0.05; § vs £, P<0.01. (PDF) [file pone.0029213.s004.pdf]

Table S2. Tumor growth rates in mice injected with siRNA-transfected C4HD cells

Treatment[1]	Mean tumor volume[2]	Growth rate[3]	Percentage of growth inhibition	
			vs. untreated cells	vs. control siRNA-treated cells
none	913 ± 216[&]	39.8 ± 6.4[&]	-	-
CONT2	1114 ± 83[£]	47.6 ± 3.3[£]	-	-
ADT	310 ± 86[§]	13.3 ± 2.9[§]	66	71

[1]Mice treated with a 40 mg s.c. MPA depot were inoculated in the flank opposite with C4HD cells transfected with 2'-O-methyl siRNA targeting IGF-IR (ADT) or with a control 2'-O-methyl siRNA (CONT2) or with untreated C4HD cells. At day 26, tumor volume and percentage of growth inhibition in tumors from mice injected with ADT siRNA transfected cells were compared to those of mice injected with control siRNA or with untreated C4HD cells. [2]Tumor volume in mm^3 ± SEM, n=5. [3]Growth rate are expressed in mm^3/day ± SEM, n = 5. § vs &, $P < 0.05$; § vs £, $P < 0.01$.
