# Supplementary material for: Small Interfering RNA Targeted to IGF-IR Delays Tumor Growth and Induces Proinflammatory Cytokines in a Mouse Breast Cancer Model
Source: PLoS One. 2012 Jan 3;7(1):e29213. doi: 10.1371/journal.pone.0029213 (PMC3250415; doi:10.1371/journal.pone.0029213)
Supplement: Table S3 — Characteristics of tumors obtained from mice injected with C4HD cells transfected with 2′-O-methyl siRNAs. 1Number of mitotic bodies per high power field (HPF). 2GM1-2 indicates 0-5 mitosis per 10 HPF; GM3 indicates >10 mitoses per 10 HPF. 3Polymorphonuclear neutrophils. (PDF) [file pone.0029213.s005.pdf]

Table S3. Characteristics of tumors obtained from mice injected with C4HD cells transfected with 2'-O-methyl siRNAs.

**Mouse group**[1]	**Mitotic Index**[2]	**Necrosis (%)**	**Fibrosis (%)**	**Infiltration**
Untreated	GM3	20-40	10	None
CONT2	GM3	40-60	20	None
ADT	GM1	40	30	Lymphocytes and PMN[3]

[1]Number of mitotic bodies per high power field (HPF). [2]GM1-2 indicates 0-5 mitosis per 10 HPF; GM3 indicates > 10 mitoses per 10 HPF. [3]Polymorphonuclear neutrophils.
